# Reliability and correlation of weight-bearing cone beam CT and Foot Posture Index (FPI) for measurements of foot posture: a test-retest study

**DOI:** 10.1007/s00256-023-04352-1

**Published:** 2023-05-03

**Authors:** Philip Hansen, Signe Brinch, Dimitar Ivanov Radev, Janus Uhd Nybing, Sanne Toftgaard, Finn Elkjær Johannsen

**Affiliations:** 1 Department of Radiology, Bispebjerg-Frederiksberg Hospital, Copenhagen, Denmark; 2 Furesø-reumatologerne, Copenhagen, Farum Denmark; 3 Institute of Sports Medicine Copenhagen, Bispebjerg-Frederiksberg Hospital, Copenhagen, Denmark

**Keywords:** Muskuloskeletal radiology, Cone beam CT, Weight-bearing CT, WBCT, CBCT, Foot Posture Index, FPI, Navicular bone, Navicular displacement, Radiology, Rheumatology

## Abstract

**Objective:**

To assess test-retest reliability and correlation of weight-bearing (WB) and non-weight-bearing (NWB) cone beam CT (CBCT) foot measurements and Foot Posture Index (FPI)

**Materials and methods:**

Twenty healthy participants (age 43.11±11.36, 15 males, 5 females) were CBCT-scanned in February 2019 on two separate days on one foot in both WB and NWB positions. Three radiology observers measured the navicular bone position. Plantar (ΔNAV_plantar_) and medial navicular displacements (ΔNAV_medial_) were calculated as a measure of foot posture changes under loading. FPI was assessed by two rheumatologists on the same two days. FPI is a clinical measurement of foot posture with 3 rearfoot and 3 midfoot/forefoot scores. Test-retest reproducibility was determined for all measurements. CBCT was correlated to FPI total and subscores.

**Results:**

Intra- and interobserver reliabilities for navicular position and FPI were excellent (intraclass correlation coefficient (ICC) .875–.997). In particular, intraobserver (ICC .0.967–1.000) and interobserver reliabilities (ICC .946–.997) were found for CBCT navicular height and medial position.

Interobserver reliability of ΔNAV_plantar_ was excellent (ICC .926 (.812; .971); MDC 2.22), whereas the ΔNAV_medial_ was fair-good (ICC .452 (.385; .783); MDC 2.42 mm). Using all observers’ measurements, we could calculate mean ΔNAV_plantar_ (4.25±2.08 mm) and ΔNAV_medial_ (1.55±0.83 mm). We demonstrated a small day-day difference in ΔNAV_plantar_ (0.64 ±1.13mm; *p*<.05), but not for ΔNAV_medial_ (0.04 ±1.13mm; *p*=n.s.).

Correlation of WBCT (WB navicular height - ΔNAV_medial_) with total clinical FPI scores and FPI subscores, respectively, showed high correlation (*ρ*: −.706; *ρ*: −.721).

**Conclusion:**

CBCT and FPI are reliable measurements of foot posture, with a high correlation between the two measurements.

**Supplementary Information:**

The online version contains supplementary material available at 10.1007/s00256-023-04352-1.

## Introduction

**Fig. 1:**
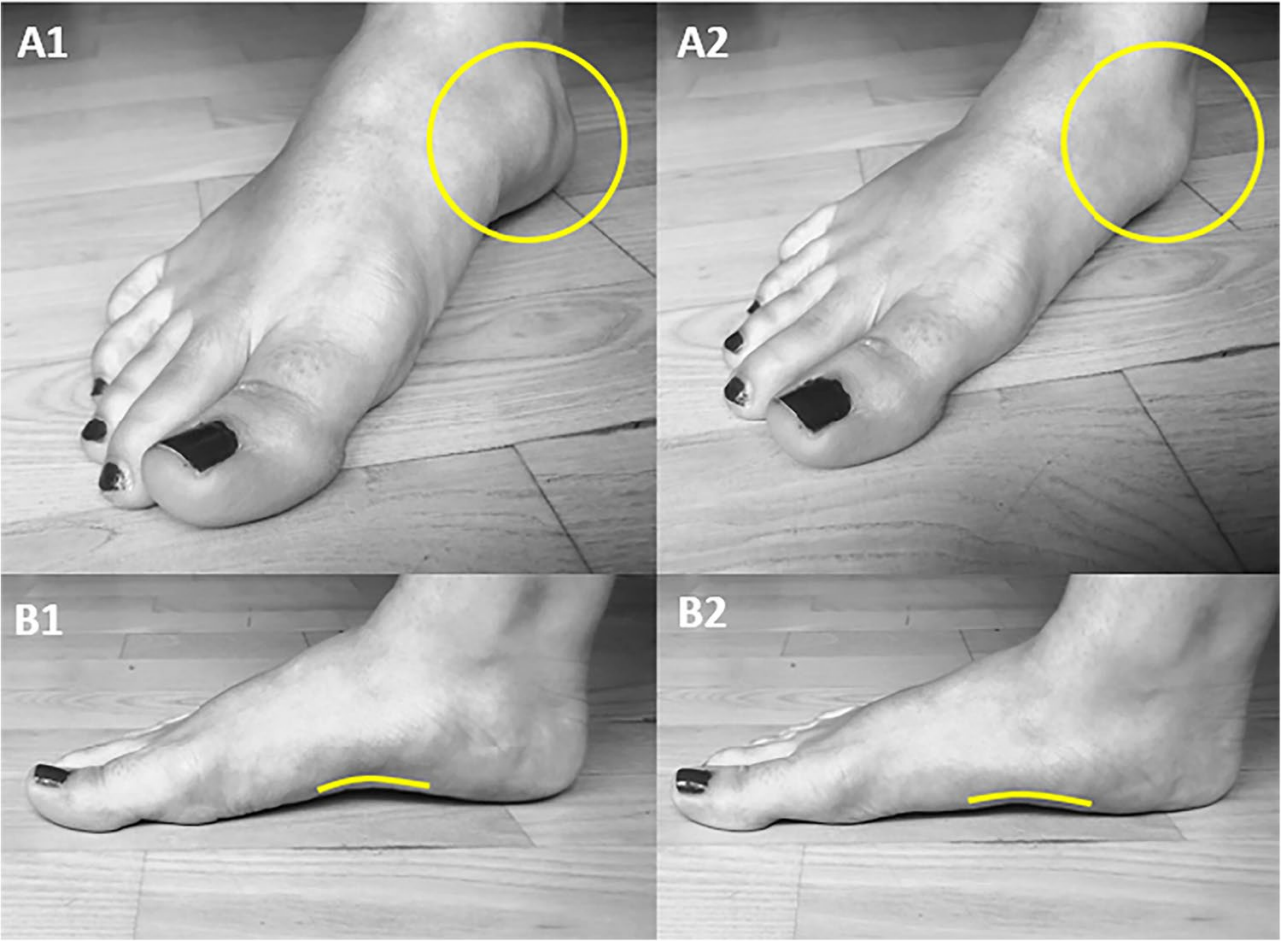
Visualisation of the medial navicular prominence in a NWB position (**A1**) and in a WB position (**A2**) and the medial longitudinal arch of a person with flexible pes planovalgus in a NWB position (**B1**) and WB position (**B2**)

The foot provides support for the body during standing and locomotion. The medial longitudinal foot arch is flexible and flattens during impact acting as an important shock absorber [1]. This ability is limited by a flat foot arch (pes planovalgus) or high foot arch (pes cavovarus) increasing the risk of injuries [2–5]. Several clinical measurements are described for assessing foot posture [6, 7], but Foot Posture Index (FPI) is the recommended assessment tool as it evaluates the posture of the whole foot [8]. FPI consists of six measurements: 3 rearfoot scores and 3 midfoot/forefoot scores all ranging from −2 to +2 resulting in a total score from −12 to +12 [6]. The 4^th^ and 5^th^ measurements of FPI assess the medial navicular prominence and the congruence of the medial internal longitudinal arch (Fig. 1). FPI has been validated against kinematic models of foot posture to satisfactorily represent both dynamic and static variables [6]. FPI can identify foot abnormalities which are speculated to increase injury risk in the lower extremity [9, 10].

Plantar displacement of the navicular bone can be measured on X-rays as a measure of foot posture [11]; however, two-dimensional X-rays are not ideal for measurement changes that occur within a 3-D anatomical system. To measure combined plantar and medial displacements, cross-sectional imaging techniques seem more adequate. With the emergence of weight-bearing cone beam CT (WBCT), it has become possible to measure 3-D displacement of bone structures imposed by static loading. An earlier study demonstrated high reproducibility of foot posture measurements on weight-bearing (WB) MRI [12]. Plantar and medial navicular positions can be measured simultaneously on WB and non-weight bearing (NWB) cross-sectional images. This allows for calculation of navicular bone displacement (ΔNAV) induced by mechanical loading in both the plantar direction (ΔNAV_plantar_) and medial direction (ΔNAV_medial_). Both WBMRI and WBCT can provide information on functional foot posture, but WBMRI takes several minutes to perform which might be strenuous and result in movement artefacts [13]. WBCT takes ~30 s and has a more precise bone depiction [14].

The purpose of this study was to investigate the reliability of measurements of both navicular bone position and displacement on WBCT and NWBCT, which are important for future research and clinical use, and to compare radiological measurements to clinical FPI assessment, which we considered to be ‘gold standard’ in terms of a clinically meaningful evaluation of foot posture.

## Materials and methods

The local ethical committee approved the study (H-18065147), and informed consent of all participants was achieved. Twenty participants (mean age: 43.11 ± 11.36, 15 males and 5 women) were recruited and scanned in February 2019. All participants included in the study were healthy without any known current or former injuries in the scanned foot or leg. Participants were included if they had a BMI<30 and were able to stand with full weight bearing on the scanned leg for at least 30 s. Participants were excluded if they were pregnant, had known arthritis or a connective tissue disease, had foot surgery or known skeletal foot disease, were unable to stand with full weight bearing on one leg, or were under 18 years of age.

WBCT and NWBCT scans were obtained from all participants twice (overall four scans) in a weight-bearing cone beam extremity CT system (Carestream OnSight 3D ® Scanner, Carestream Health, Rochester NY, USA). Scan parameters were 90 kV and 6 mA with an isotropic voxel size of 0.26×0.26×0.26 mm. The effective dose was maximally 40 μSv per scan [14, 15], but usually below this approximately 12–15 μSv per scan, which is much less than conventional CT [14–16]. In total, each participant was exposed to a maximum of 160 μSv. According to health authority data, the background risk of sustaining cancer during the total life span is ~25%. Exposure to 1 Sv increases the risk by ~5%. Hence, an exposure of 160 μSv would increase the theoretical background risk of cancer by 0.00016 Sv * 5% = 0.0008%. This risk is deemed negligible as an ethical consideration.

The cone beam CT scanner allows weight-bearing scans and, due to the scanner design, it is possible to walk directly into the scanner gantry, as the gantry door easily opens. During scanning only the scanned extremity is in the scanner while the non-scanned extremity can rest outside the gantry or on top of the gantry depending on scan type [14, 15]. During WB scans of the foot, a platform is placed in the scanner gantry, which secures that the foot is within the field of view during scanning. The participants simply step in, on top of the platform.

For test-retest repeatability, participants were scanned twice, on two different days, in both WB and NWB positions, approximately at the same time of day, with an average interval of 3 days between scanning sessions. In both sessions, the foot was scanned with the knee positioned in full extension while standing with the foot on a horizontal supporting platform surface. The non-supporting leg was placed outside the gantry.

The WBCTs were obtained by asking the participants to place 80% of their body weight on the foot inside the scanner. For the NWBCTs, participants were asked to place only 20% of their body weight on the foot inside the scanner. To ensure this subjective weight distribution, a scale was placed under the foot outside the gantry to monitor the load, compared to their previously measured full body weight.

For practical reasons, we used three radiological observers: two experienced musculoskeletal radiologists and a junior doctor. All observers were specifically trained in these measurements beforehand, and independently performed measurements on both WBCT and NWBCT images. Images were measured once by the musculoskeletal radiologist and twice by the junior doctor with an interval of 4 weeks to obtain both inter- and intraobserver reproducibilities. One senior radiologist measured the plantar displacement of the navicular bone while the other senior radiologist measured the medial displacement of the navicular bone.

**Fig. 2:**
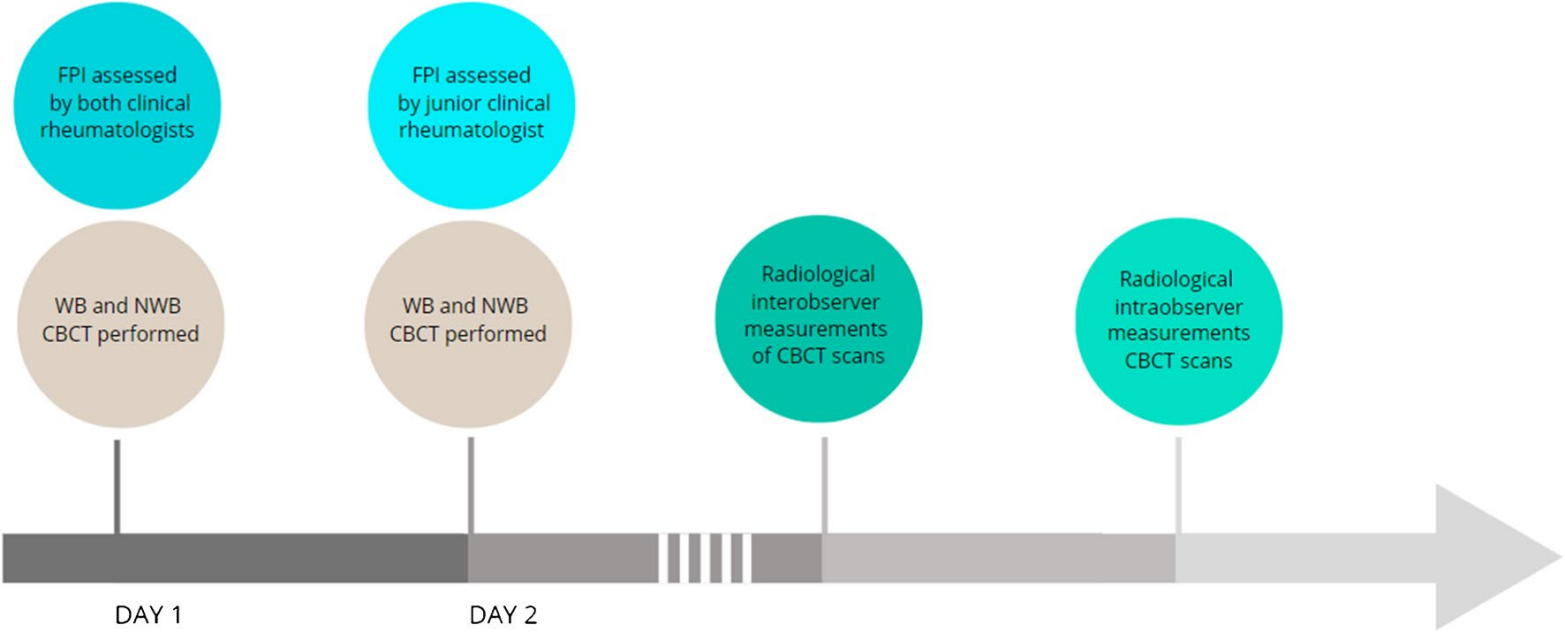
Flowchart showing workflow of scans, clinical FPI assessment, and radiological measurements

A senior rheumatologist and a junior rheumatologist independently performed clinical measurements of the FPI on all 20 participants on the same day as the first CBCT. The junior rheumatologists repeated the clinical assessment on the same day the second CBCT was performed (Fig. 2). The FPI consists of six measurements scoring −2 to +2 resulting in a total score from −12 to +12. Three rearfoot scores: (1) talar head palpation, (2) curves above and below the lateral malleoli, (3) calcaneal valgus/varus; and 3 midfoot/forefoot scores: (4) talo-navicular congruence (concave or bulging), (5) medial arch height, (6) forefoot abduction/adduction [6]

### Image plane correction

**Fig. 3:**
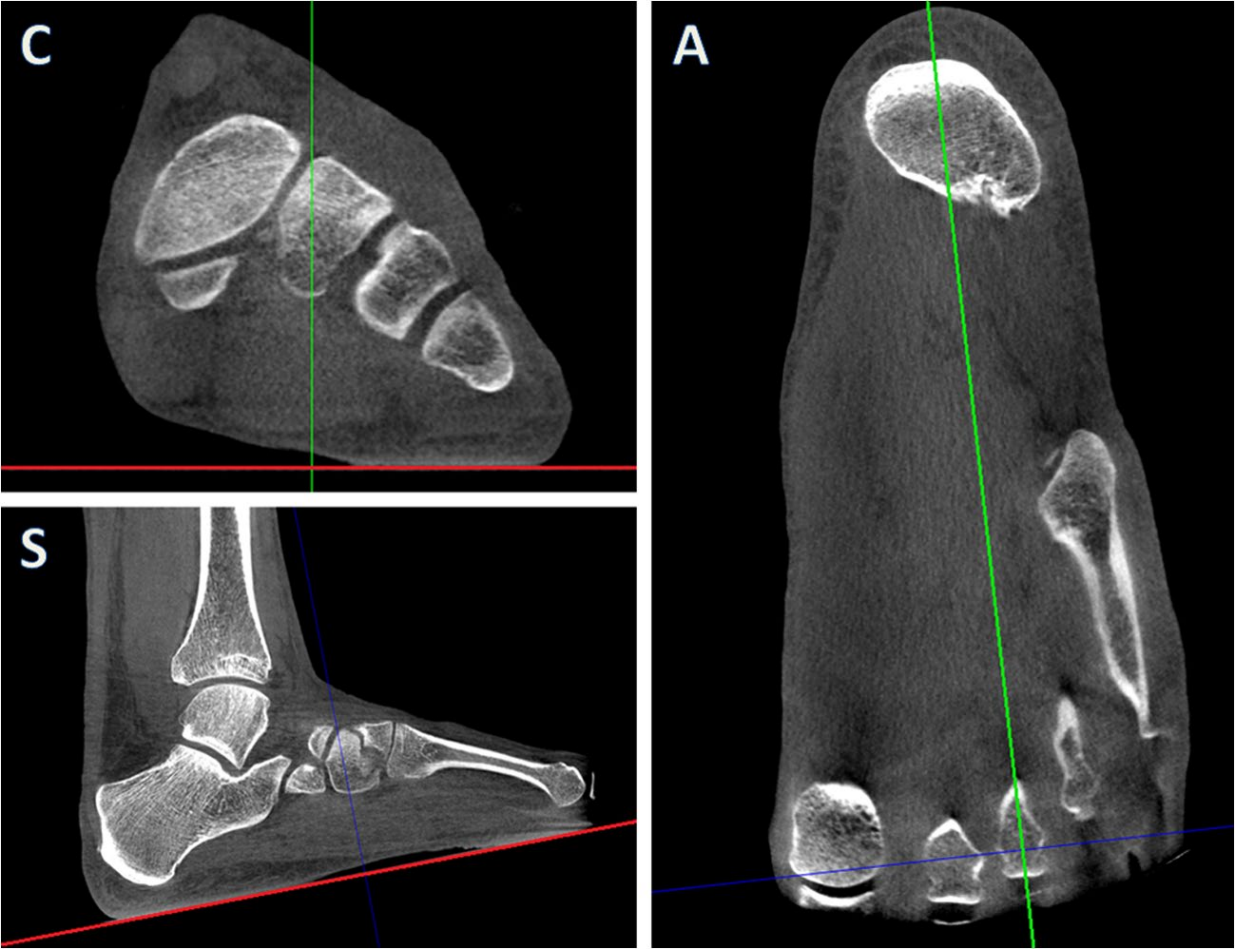
Adjustments of the mulitplanar image planes prior to measurements. On the coronal (C) and sagittal images (S), the axial reference plane (red lines) were adjusted to align with the underlying carbon plate. On the axial image (A), the sagittal reference plane (green line) was adjusted to intersect the head of the 3^rd^ metatarsal bone and the centre of the calcaneal bone

To ensure standardised image planes prior to measurements, the original image dataset was image plane adjusted by a prespecified procedure in a PACS multiplanar reconstruction (MPR)-module to standardise image planes before measurements. The first step was to adjust the axial plane to align with the underlying horizontal supporting platform on both the coronal and sagittal images (Fig. 3C and S). The second step was to adjust the sagittal plane on the axial image. The sagittal plane was adjusted using a method inspired by the so-called foot and ankle offset (FAO) which is a measurement method by Lintz et al. [17] that uses the WB points of the 1^st^ and 5^th^ metatarsal heads as well as the centre of the calcaneal tuberosity as bony landmarks to determine a reference sagittal plane. In our study, the sagittal plane (Fig. 3A) was adjusted to intersect the 3^rd^ metatarsal head and running approximately straight through the centre of the calcaneal tuberosity.

### Image analysis

**Fig. 4:**
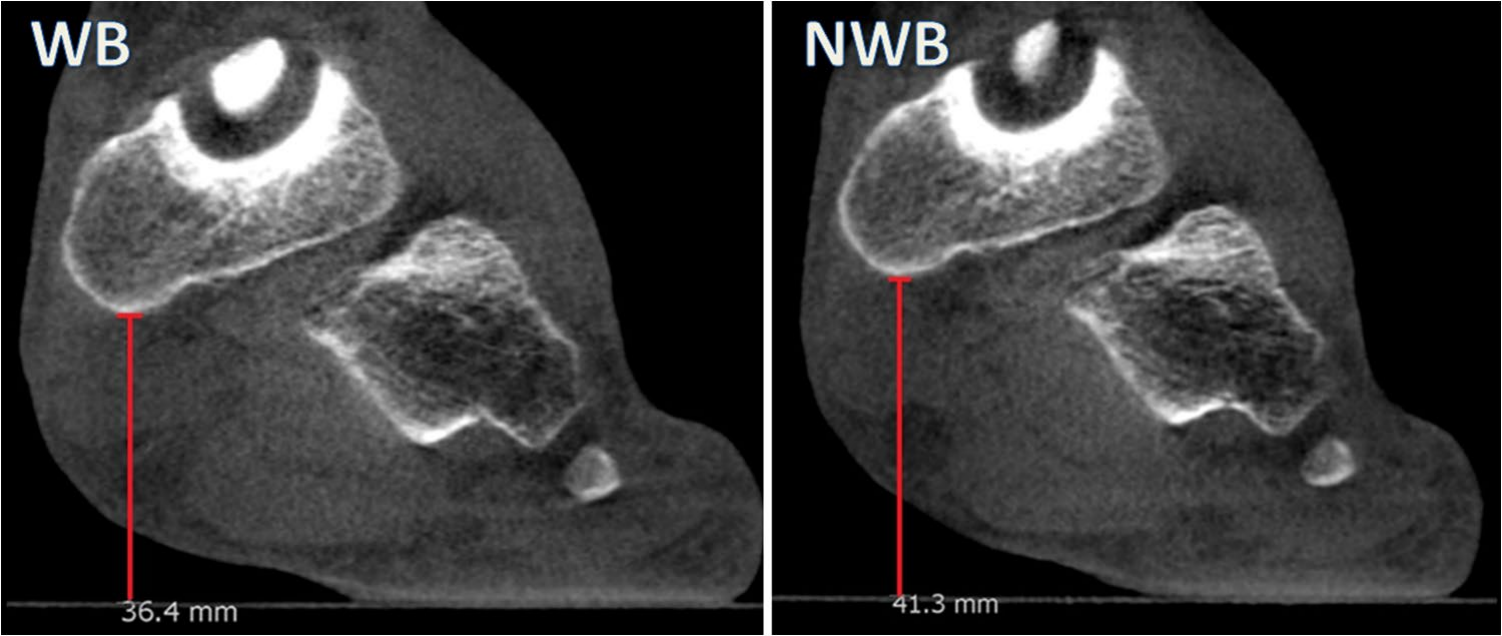
Measurement of the navicular plantar position on the same participant in WB and NWB positions. The plantar displacement was measured as the interposed space between the most caudal osseous contours of the navicular bone to the underlying carbon plate

Using the saved standardised image dataset, all images were again assessed in the MPR module by measuring the plantar and medial displacements of the navicular bone between WB and NWB positions. Plantar displacement was measured on the coronal plane, on the slice with the most caudal osseous landmark of the navicular bone, perpendicular to the underlying platform surface (Fig. 4). This platform was in our experience always clearly defined on WBCT images providing a solid reference of measurement.

**Fig. 5:**
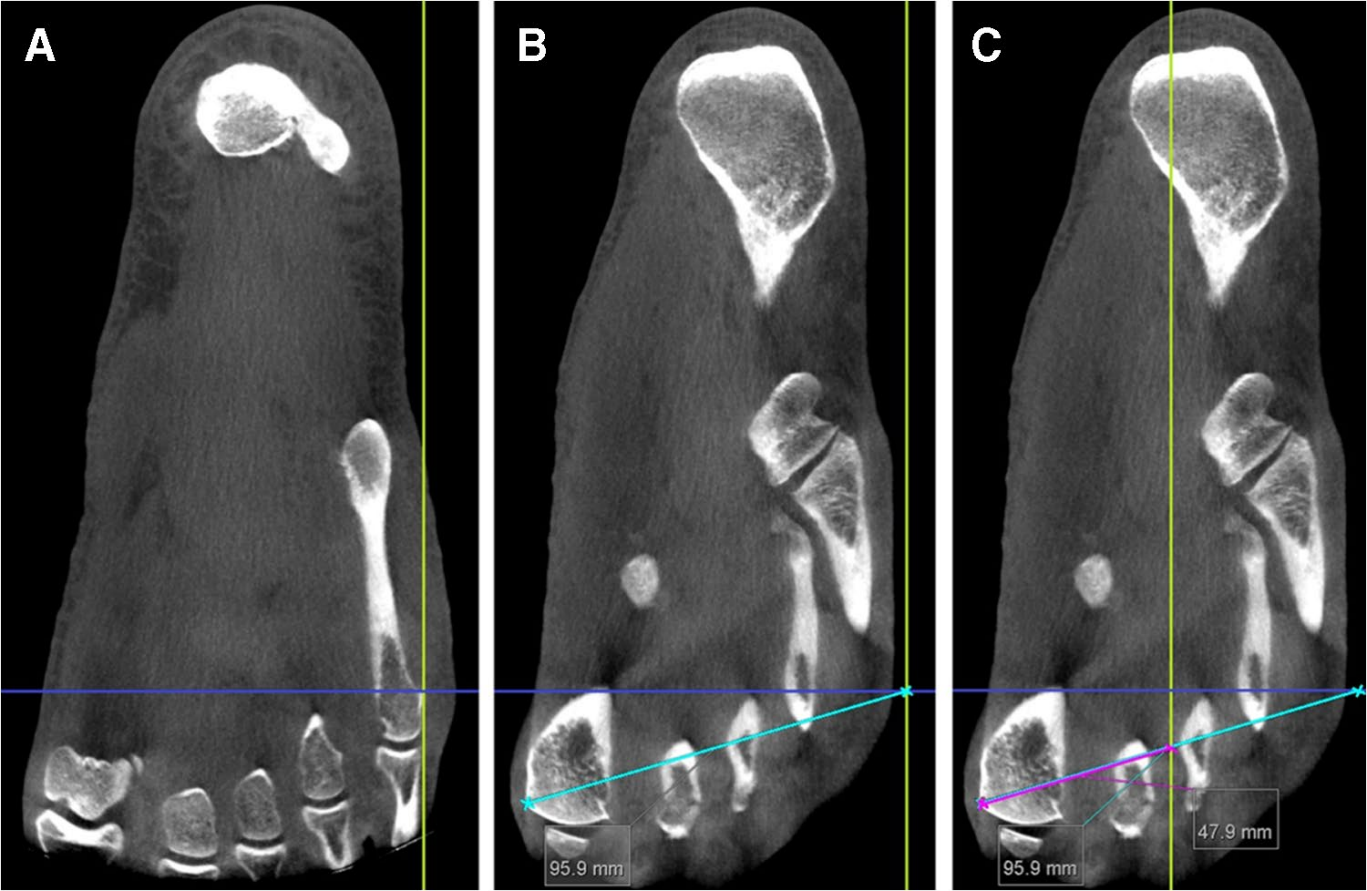
Measurements of the medialisation of the navicular bone. In (**A**), the most lateral part of the 5^th^ metatarsal head was found and identified by placing the intersection of the saggital reference plane (yellow line) and coronal reference plane (blue line) tangentially to this point. The slice with the most medial part of the head of the 1^st^ metatarsal bone was identified (**B**) and a reference line (turquoise) was drawn from this medial point to the lateral point. The centre point of the reference line was identified (purple line) (**C**) and the sagittal plane was moved to intersect this centre point

**Fig. 6:**
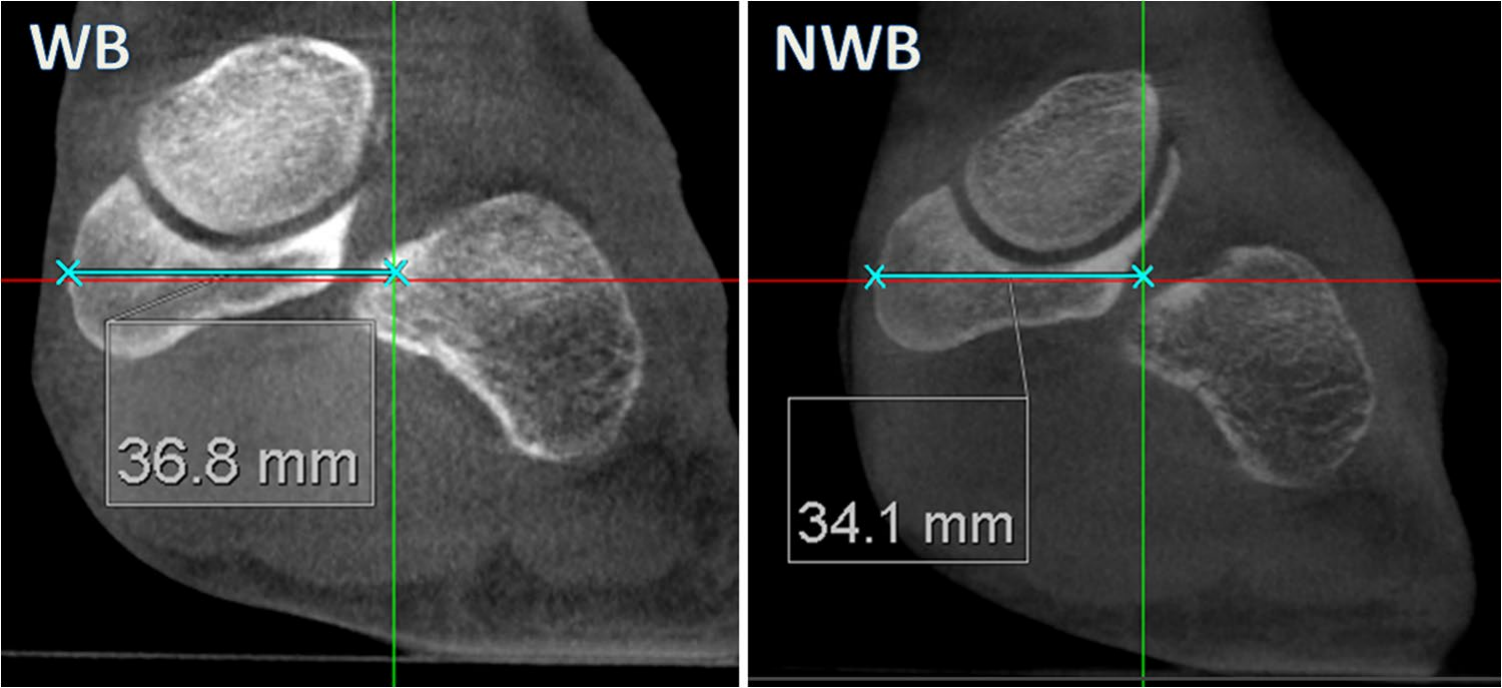
Measurement of the navicular medialisation in WB and WNWB positions respectively on the coronal plane.The medialisation is measured as the interposed distance between the most medial osseous contour of the navicular bone to the predefined midpoint of the foot (sagittal reference plane as in Fig. 5C, green)

The medialisation of the navicular bone was measured on the axial plane with a method, again, inspired by the FAO [17]. The first step was to identify the bony landmarks of the heads of the 1^st^ and 5^th^ metatarsal bones. The slice with the most lateral part of the head of the 5^th^ metatarsal bone visible was identified, and the intersection of the sagittal plane (yellow line, Fig. 5A) and the coronal plane (blue line, Fig. 6A) was placed tangentially to this point. The slice with the most medial part of the head of the 1^st^ metatarsal bone was identified, while the sagittal plane (yellow line, Fig. 5B) was kept at the point of the head of the 5^th^ metatarsal bone. After the 1^st^ and 5^th^ metatarsal landmarks were identified, a perpendicular line (turquiose line, Fig. 5B) was drawn between these two points. The centre of the perpendicular line was found by dividing the length of the measurement in half and measured with a new measurement line (pink line, Fig. 5C), overlying the perpendicular line. The saggital plane in the MPR module was then moved to the centre point, corresponding to the ‘middle’ of the foot (yellow line, Fig. 5C)

The navicular medial displacement was measured on the coronal plane as the perpendicular distance from the most medial osseous navicular landmark to the coronal slice centred between the first and fifth metatarsal bones identified as stated above (sagittal plane (green sagittal line corresponding to the yellow sagittal line in Fig. 5C).

FPI is a clinical assessment of the foot posture in standing. The higher the score, the lower and more medialized the navicular bone is positioned. For a comparison between FPI and CBCT, we therefore chose to use CBCT-measured height position of the navicular bone in weight bearing and subtracted the delta medialisation (change from non-weight bearing to weight bearing), expecting an inverse correlation.

### Statistics

Descriptive statistics were used to describe quantitative and qualitative data. All measurements on CBCT were continuous and determinations of FPI scores were ordinal (−12 to +12). Based on a former study [18], a standard deviation (SD) of 1.1 mm between test-retest measurements was used to calculate the required minimum number of participants to 19 at MIREDIF 1 mm, alpha 5%, beta 20%. Intraclass correlation coefficients (ICC) based on analysis of variance (ANOVA) were calculated. ICC estimates and their 95% CI were calculated using SPSS (IBM Corp., version 25 for Macintosh), based on mean rating (*k*=2), consistency, two-way mixed-effects model for interobserver reproducibility and mean rating (*k=*1), absolute agreement, two-way mixed-effects model for intraobserver reproducibility [19–21]. According to Rosner [22], the inter- and intraobserver reproducibility values of 0.4, 0.4< to <0.74, and > 0.75 are indicative of poor, fair to good, and excellent reproducibility, based on the 95% CI of the ICC estimate. For assessment of systematic differences between repeated measurements, a paired Student’s *t*-test was calculated. Minimal detectable change (MDC; ≈95% probability level), analogous to the one-sided Bland-Altman (BA) limits of agreement (LOA), was calculated as a measure of agreement between inter- and intraobserver measurements. These metrics were applied to both the measurements of static bone position in WB and NWB as well as for ΔNAV. For clarity on intra- and interobserver reproducibility, results are reported for day 1 measurements only. Sample characteristics were evaluated by calculation of the mean and SD. To obtain a best weighted estimate of ΔNAV between WB and NWB in which we wanted to average out individual observer variation to assess the precision of the overall method, we calculated the average of the ΔNAV_day1_ and ΔNAV_day2_ for the junior observer and senior observer as a total mean. Spearman’s correlation (*ρ*), a nonparametric linear correlation, was used for assessment of correlation between WBCT and clinical FPI measurements. According to Mukaka [23], the correlation values of ±.00 to ±0.30, ±0.30 to ±0.50, ±0.50 to ±0.70, ±0.70 to ±0.90, and ±0.90 to ±1.00 are indicative of negligible, low, moderate, high, and very high correlations.

## Results

The intra- and interobserver reliabilities for CBCT assessments of navicular position and FPI measurements were “excellent” (ICC .875–.997). Intraobserver reliability for navicular height and medial position was particularly high (ICC .0.967–1.000) and interobserver reliability also ‘excellent’ (ICC: .946–.997). MDC were <1 mm and <3 mm for plantar and medial positions, respectively. In the medial position, a statistically significant difference (systematic error) was found between the observers (0.47±0.92 mm; *p*<0.05) (table A3, appendix A).

In terms of reproducibility, no heteroscedasticity was found in the corresponding BA plots (intraobserver, fig. B1–B4, appendix B) (interobserver, fig. C1–C8, appendix C). Outliers were identified in all but two of eight BA plots (appendices B and C).

Concerning the calculated displacement (ΔNAV)*:* The interobserver reliability of ΔNAV_plantar_ was excellent (ICC .926 (.812; .971); MDC 2.22), ΔNAV_medial_ was fair-good (ICC .452 (.385; .783); MDC 2.42 mm) (Table 1).

**Table 1:** Delta difference between displacements between days

Intraobserver		
	Plantar displacement	Medial displacement
*p***-**value	*	n.s.
Mean	4.17	1.67
SD	2.17	.92
Mean_diff_	.53	.06
SD_diff_	1.06	1.46
SEM	.75	1.03
MDC	2.07	2.87
ICC	.930(.802;.973)	.381(-.642,.759)
Interobserver		
Delta difference of displacement day 1 vs day 2		
	Plantar displacement	Medial displacement
*p***-**value	*	n.s.
Mean	4.25	1.55
SD	2.08	.83
Mean_diff_	.64	.04
SD_diff_	1.13	1.23
SEM	.80	.87
MDC	2.22	2.42
ICC	.926 (.812;.971)	.452 (.385;.783)

*p*-value: *= *p* ≤0.05, n.s.= *p* > .05 (non-significant), *Mean* of measurements in mm, *SD* standard deviation in mm, *Mean*_*diff*_ mean difference of measurements in mm, *SD*_*diff*_ difference of standard deviation in mm, *SEM* standard error of the mean in mm, *MDC* minimal detectable change in mm, *ICC* intraclass correlation coefficient

**Fig. 7:**
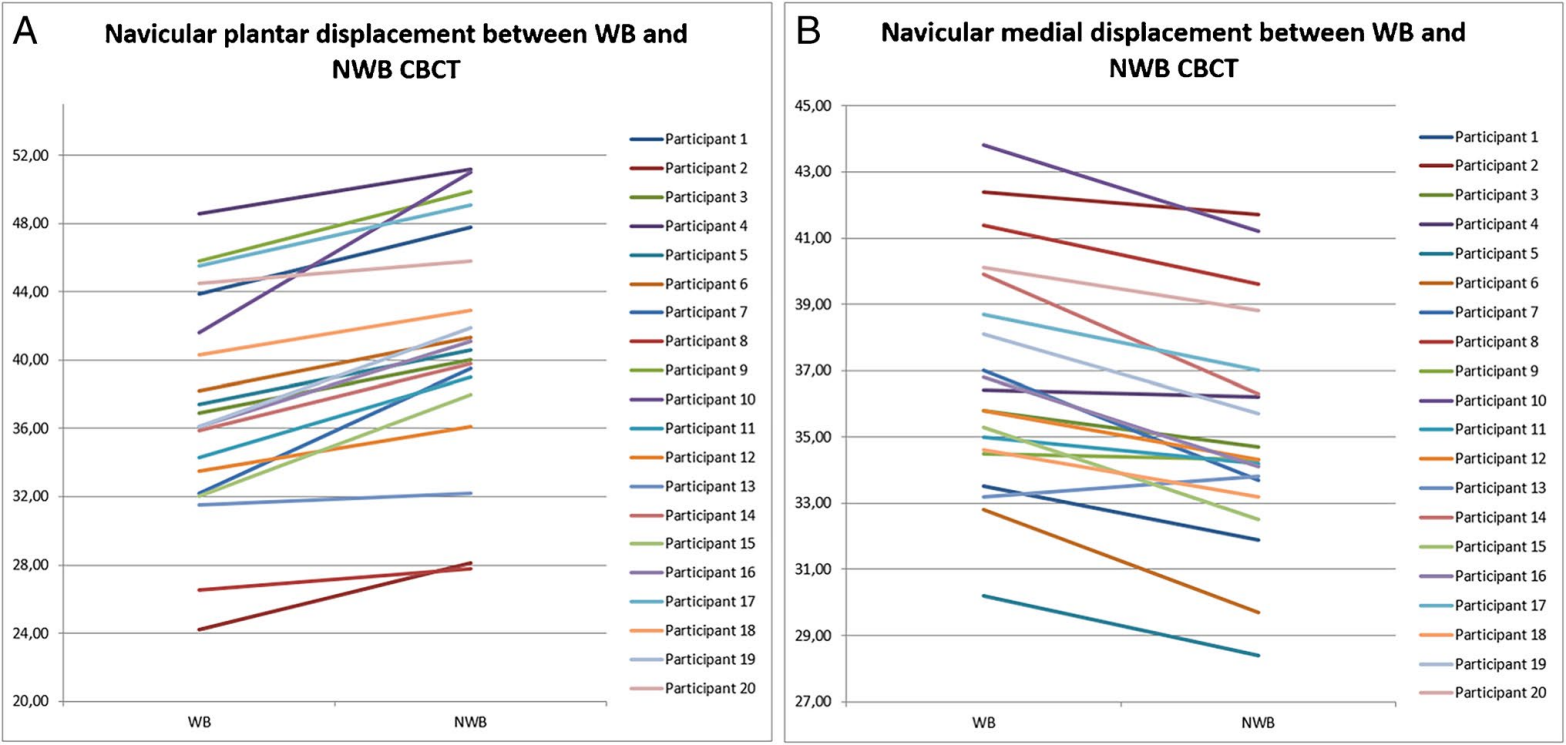
**A** The navicular plantar displacement (mm) between WB and NWB positions of all twenty participants. **B** The navicular medial displacement (mm) between NWB and WB positions of all twenty participants

When averaging all observer measurements on both days and calculating the ΔNAV, we could demonstrate a plantar displacement (ΔNAV_plantar_) 4.25±2.08 mm and a medial displacement (ΔNAV_medial_) 1.55±0.83 mm; *p*<.0001. In Fig. 7, we illustrate navicular bone displacement for all participants (plantar and medial). We demonstrated a small but significant difference (0.64 ±1.13mm; *p*<.05) between day 1 and day 2 for ΔNAV_plantar_, but not for ΔNAV_medial_ (0.04 ±1.13mm; *p*=n.s.) (table A3, appendix A) (Table 1).

For clinical FPI measurements, intraobserver and interobserver reproducibilities were found to have ‘excellent’ ICCs (ICC .875 (.684; .950); ICC .875 (.683; .950), respectively; Table A4, appendix A). Interobserver reproducibility was measured between the junior and the senior rheumatologists. A small bias was found between the two observers (*p*<.05) MDC of 3.55 and 3.76 and SEM of 1.36 and 1.28 mm were found for intraobserver and interobserver measurements, respectively (table A4, appendix A). No heteroscedasticity or outliers were identified in corresponding BA plots (figure E1–E2, appendix E).

**Fig. 8:**
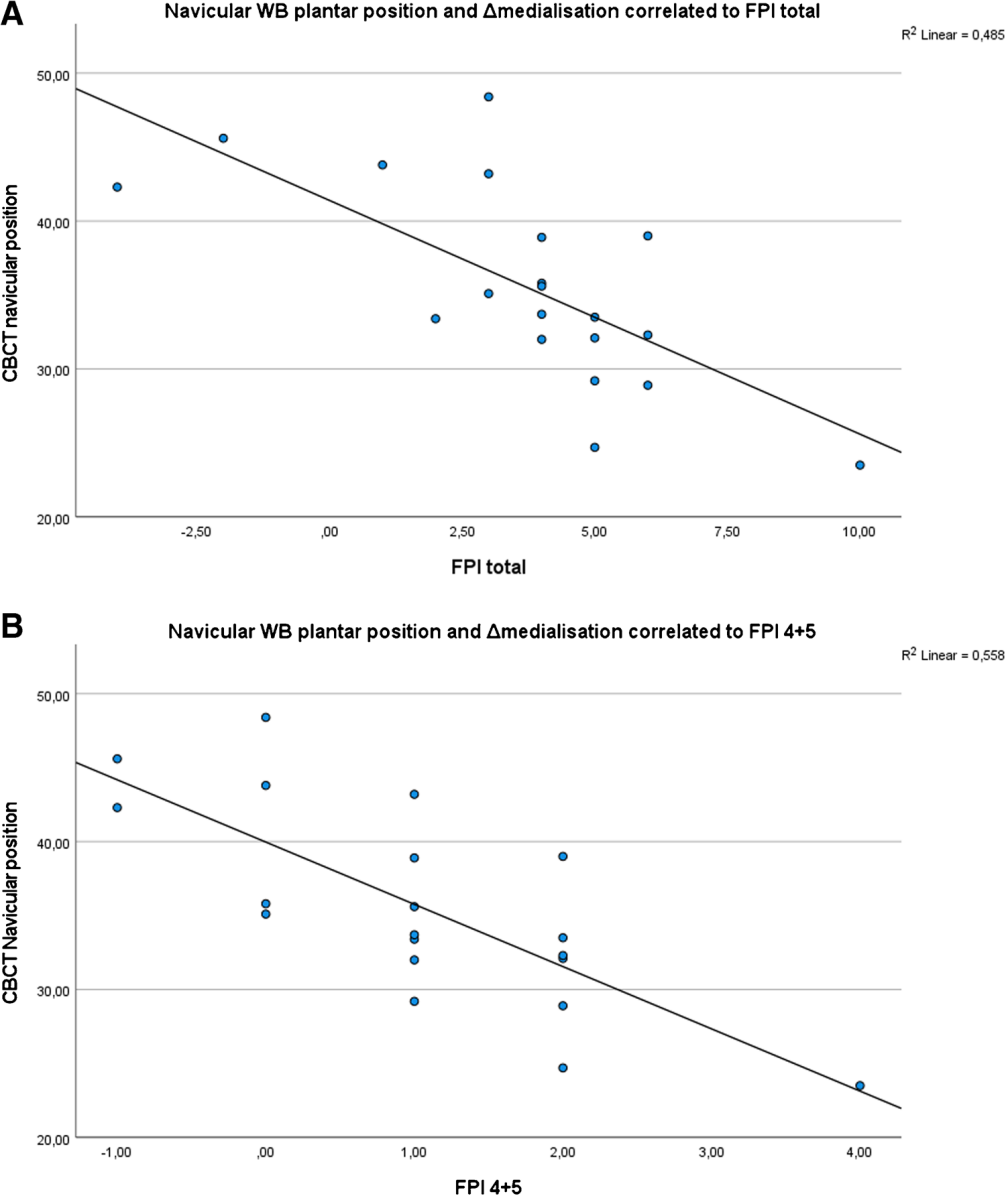
**A** Correlation between CBCT navicular position and FPI total. **B** Correlation between CBCT navicular position and FPI 4+5

In Fig. 8A-B, we illustrate the correlation between CBCT (WB plantar height minus ΔNAV_medial_) and the clinical FPI measurements of the senior rheumatologist for each participant. We correlated to the total FPI score, as well as to the sub scores FPI 4+5. This showed a ‘high’ negative correlation (*ρ*: −.706; *ρ*: −.721) (Table 2).

**Table 2:** Spearman’s correlation (*ρ*) of WBCT and clinical FPI measurements

	FPI total score	FPI 4+5 score
WB plantar position - ΔMedialisation	*ρ* = −0.706**	*ρ* = −0.721**

**Correlation is significant at the 0.01 level (2-tailed)

## Discussion

In the present study, we demonstrated good test-retest repeatability and measurement reproducibility of CBCT measurements of the navicular bone position in both WBCT and NWBCT. The main finding was that the measurement method of navicular bone position on CBCT, e.g. plantar and medial positions, was reproducible with no clinically significant bias between days. Furthermore, the clinical assessment of FPI showed high reproducibility.

Regarding the inter- and intraobserver reproducibilities of the radiological measurements, our results showed ‘excellent’ reliability and agreement in particular represented by almost perfect intraobserver reliability and no systematic difference between repeated measurements for navicular position. The ability to repeatedly measure a given navicular position on unchanged images may not be surprising due to the clear delineation of bone margins and other points of reference on CBCT and the precise measuring methods described. We would consider ΔNAV_plantar/medial_ a more difficult assessment in terms of reproducibility since variation stemming from two position measurements will tend to increase variation even more so between days. Nonetheless, we found excellent between-day reliability for ΔNAV_plantar_ while ΔNAV_medial_ only displayed ‘fair to good’ results, which is obviously not ideal. We can only speculate whether ΔNAV_medial_ is more sensitive to small within-subject variation between days than ΔNAV_plantar_. It can be speculated that stress relaxation of the medial arch is a factor that may introduce between-day variation but any reason for ΔNAV_medial_ being more sensitive to such variation that ΔNAV_plantar_ remains uncertain. This is coherent with former reproducibility studies on the navicular bone displacement, concerning both clinical navicular bone drop measurements [24–27] and measurements done on both cross-sectional imaging [18] and X-rays [28]. Overall, the measurement method in the present study is deemed highly reproducible in particularly so to determine specific navicular position, which could be quite relevant in many clinical situations, e.g. to evaluate the effect on static foot posture following correction, e.g. by foot surgery or insoles. Our results for navicular position certainly support general applicability of the measurement technique.

For ΔNAV_plantar_, we found a small difference between days (Table 1) of 0.64 mm (±1.13 mm; *p*<.05). This difference is most likely due to a day-to-day variance in foot posture and/or a change in scanning method; however, we could only depict such a small difference due to the very high reproducibility of the measurement methods, and we do not find that the magnitude of difference represents a concern that should discourage use of the method.

Similar findings have previously been observed using WB and NWB MRI by which especially between-day reproducibility in supine non-WB position was found somewhat unreliable [18]. Our results of the plantar ΔNAV_plantar_ resemble an earlier study by Eichelberger et al. [29], in which the intra- and interday values of the navicular drop (ΔNAV_plantar_) were found to be 1.1 mm (ICC .97) and 2.3 mm (ICC .87) corresponding to our results. The studies, however, are not directly comparable, as Eichelberger et al. used skin markers in their clinical measurements. The ICCs of the ΔNAV_medial_ were found to be ‘poor’ to ‘fair’, which could in part be related to the homogeneity of the sample with data points within a very small range of −0.6 to 3 mm since homogenous samples all other factors being equal will yield poorer values of ICC compared to heterogenous samples [30]. The corresponding BA plots for ΔNAV_medial_ show no heteroscedasticity, only a single outlier and a very small mean_difference_ between days of 0.04 mm, suggesting that the test-retest results of the medial displacement are more robust that just indicated by ICC values. Nonetheless, further improvement of the method to increase reliability of ΔNAV_medial_ is desirable.

Regarding FPI, a recent study of the reliability by Aquino et al. [8] concluded that inter- and intra-reliability studies of the FPI had conflicting results, varying from ‘poor’ to ‘good’ results using weighted Kappa. Our results of inter- and intraobserver reliability of the FPI measurements demonstrated ‘excellent’ reliability by ICC. This corresponds to [24–27, 31] and/or surpasses [32, 33] former reproducibility studies on clinical FPI measurements. FPI evaluates foot posture in several planes which could explain a weak correlation observed between FPI and navicular drop on X-rays [34]. Because CBCT allows assessment the foot in all planes, we speculate that correlation between FPI and CBCT is higher than between FPI and X-rays. However, this assumption requires further investigation.

Even though we found ‘excellent’ reliability, we a small statistically significant bias between observers was observed. We deemed this not clinically important due to the interobserver SEM being only 1.3 mm corresponding to one point in the FPI assessment, which would not skew results from e.g. pronated to supinated or vice versa.

When correlating the clinical FPI assessment with the measurements of the CBCT, we found ‘high’ correlation. This is in agreement with the results of Patel et al. [35] who also used WBCT and FAO. They concluded that FPI is an accurate clinical measurement tool and that clinical FPI measurements correlate well with measurements from WBCT scans. The results of Patel et al. are state of the art as they used semiautomatic 3D biometrics to calculate FAO on WBCT scans. However, we believe that our method of measuring ΔNAV in the commonly available PACS system at radiology departments could have a broader impact since it is generally applicable for clinical investigation of foot alignment in particularly since we also found a ‘high’ correlation between clinical and radiological assessments, which would indicate clinical relevance of WBCT in terms of measuring foot posture.

## Limitations and perspectives

We could not strictly control the day-to-day difference stemming from biological factors such as stress relaxation of the connective tissues in the medial plantar arch, which in theory could lead to some unknown degree of ‘flattening’ of the plantar arch. Slight arch flattening could occur during the scanning, but since the total scan time including technical planning of the scan is in the order of approximately 2 min, we do not expect stress relaxation during scanning to significantly impact measurements. However, participants were not scanned on precisely the same time of day, and we cannot account for any differences in stress relaxation induced by differences in the amount of standing or walking occurring before scanning. To our knowledge, it is not known how much normal daily function affects foot bone posture by stress relaxation.

The results of this article are based on a cohort consisting of healthy subjects, and we cannot be sure that our reproducibility results are directly applicable to other patient cohorts. However, to our knowledge, no studies have demonstrated that foot posture changes with the most common overuse injuries in the lower limb, such as Achilles tendinopathy and plantar fasciitis, although it is speculated these injuries are partly caused by a preexisting abnormal foot posture [2–5]. In a dynamic cadaveric and invasive bone pin study of foot bone positions by Nester et al. [36], it was concluded that variation in foot kinematics and joint movements in the foot is both considerable and universally present. The authors conclude that one should avoid attempts to make an ‘ideal’ model for all feet, but rather identify patient-specific models of foot kinematics, as there might be different ways of joint motion depending on differences in foot construction, different ways of landing (heel landing, midfoot landing, forefoot landing), propulsion (outtoeing, intoeing), mechnoreception and muscle function.

We believe that the use of both the clinical FPI measurements and the WBCT could aid in a more individualized, yet detailed, anatomical and functional approach to further understand foot function and evaluate to which degree abnormal foot posture is a risk factor for lower limb injuries. Ideally, the evaluation by CBCT should evaluate the intricate displacement of multiple tarsal bones to gain further understanding of foot function. However, as our results show, this will likely be challenging since simply quantifying navicular bone displacement in a 3D coordinate system is complex.

Currently, the availability of WBCT is limited but likely this imaging modality will become more widespread as indicated by steadily increasing amounts of musculoskeletal research using WBCT not least in assessment of the foot [37].

In perspective, we suggest that future efforts are aimed at providing even more reliable scanning protocols for standardised control of foot posture during WBCT. This will hopefully help to further evaluate the intricate influence of different loading patterns and correctional procedures on the foot posture, e.g. by precisely monitoring magnitude of mechanical loading during WBCT as well as highly standardised radiological measurement procedures including standardised image plane correction.

## Conclusion

We have shown a high degree of correlation between WB CBCT measurements of navicular bone displacement and FPI. Since FPI can be regarded as ‘gold standard’ in terms of clinical relevance, our results seem to validate future use of CBCT in assessment of foot posture bringing its usefulness in 3D evaluation of foot posture forward. Reproducibility of both methods remained high particularly so for WBCT measurement of navicular height and medial position while ΔNAV_medial_ showed only fair to good reliability. Future efforts should continue to refine CBCT protocols to reduce sources of measurement variation maximally if measurement of ΔNAV is intended. For mere determination of navicular position, the method is deemed highly precise.

### Supplementary information

ESM 1 (DOCX 226 kb)
