# Supplementary material for: Reliability and correlation of weight-bearing cone beam CT and Foot Posture Index (FPI) for measurements of foot posture: a test-retest study
Source: Skeletal Radiol. 2023 May 3;52(12):2387–97. doi: 10.1007/s00256-023-04352-1 (PMC10582147; doi:10.1007/s00256-023-04352-1)

**SUPPLEMENTARY MATERIAL**

**APPENDIX A**

| Table A1 – Difference between WB vs NWB for day 1 | | |
| --- | --- | --- |
|  | **Navicular plantar displacement** | **Navicular medial displacement** |
| p-value | *** | *** |
| MDC | 4.08 | 2.20 |
| Mean_diff_ | 3.91 | 1.70 |
| SD_diff_ | 2.08 | 1.12 |
| p-value: ****= p <0.001, MDC: minimal detectable change in mm. Mean_diff_: mean of measurements in mm, SD: standard deviation in mm, SD_diff_ :standard deviation in mm | | |

| **Table A2 – Intraobserver reproducibility of radiological measurements** | | | | |
| --- | --- | --- | --- | --- |
|  |  | | **DAY1 1^st^ round vs 2^nd^ round** | |
|  | **Plantar position** | | **Medial position** | |
|  | **WBCT** | **NWBCT** | **WBCT** | **NWBCT** |
|  |  |  |  |  |
| **p-value** | n.s. | n.s. | n.s. | n.s. |
| **Mean** | 37.22 | 41.15 | 36.59 | 34.95 |
| **SD** | 6.47 | 6.82 | 3.69 | 3.19 |
| **Mean_diff_** | .05 | .01 | .18 | .12 |
| **SD_diff_** | .29 | .17 | 1.30 | 1.53 |
| **SEM** | .20 | .12 | .92 | .90 |
| **MDC** | .57 | .33 | 2.54 | 2.99 |
| **ICC** | 1.000(.999;1.000) | 1.000(1.000;1.000) | .967(.918;.987) | .946(.864;.979) |
|  |  | | **DAY 1 vs DAY 2** | |
|  | **Plantar position** | | **Medial position** | |
|  | **WBCT** | **NWBCT** | **WBCT** | **NWBCT** |
| **p-value** | n.s. | n.s. | n.s. | n.s. |
| **Mean** | 37.17 | 41.34 | 36.68 | 35.01 |
| **SD** | 6.44 | 6.72 | 3.52 | 3.22 |
| **Mean_diff_** | 0.16 | 0.37 | 0.18 | 0.12 |
| **SD_diff_** | 1.13 | 0.94 | 1.30 | 1.53 |
| **SEM** | 0.80 | 0.66 | 0.92 | 1.08 |
| **MDC** | 2.21 | 1.84 | 2.54 | 2.99 |
| **ICC** | .933(.981;997) | .995(.986;998) | .967(.918;.987) | .946(.864;.979) |
| p-value: n.s.= p > .05 (non-significant), Mean: mean of measurements in mm, SD: standard deviation in mm, Mean_diff_: mean difference of measurements in mm, SD_diff_ : difference of standard deviation in mm, SEM: standard error of the mean, MDC: minimal detectable change, ICC: Intraclass correlation coefficient | | | | |

| **Table A3 – Interobserver reproducibility of radiological measurements** | | | | |
| --- | --- | --- | --- | --- |
|  | **Navicular plantar position** | | | |
|  | **DAY1** | | **DAY 2** | |
|  | **WBCT** | **NWBCT** | **WBCT** | **NWBCT** |
|  |  |  |  |  |
| **p-value** | n.s. | n.s. | n.s. | n.s. |
| **Mean** | 37.34 | 41.27 | 37.08 | 41.65 |
| **SD** | 6.33 | 6.74 | 6.31 | 6.55 |
| **Mean_diff_** | .18 | .23 | .02 | .25 |
| **SD_diff_** | .76 | .71 | .94 | .71 |
| **SEM** | .54 | .50 | .67 | .50 |
| **MDC** | 1.48 | 1.39 | 1.85 | 1.40 |
| **ICC** | .996(.991;.999) | .997(.993;.999) | .997(.993;.999) | .997(.993;.999) |
|  | **Navicular medial position** | | | |
|  | **DAY1** | | **DAY 2** | |
|  | **WBCT** | **NWBCT** | **WBCT** | **NWBCT** |
|  |  |  |  |  |
| **p-value** | ** | * | n.s. | n.s. |
| **Mean** | 36.36 | 34.83 | 36.41 | 34.84 |
| **SD** | 3.42 | 3.43 | 3.58 | 3.04 |
| **Mean_diff_** | .81 | .47 | .35 | .22 |
| **SD_diff_** | 1.02 | 0.92 | 1.19 | 1.41 |
| **SEM** | .72 | .65 | .84 | 1.00 |
| **MDC** | 2.00 | 1.81 | 2.33 | 2.77 |
| **ICC** | .978(.943;.991) | .982(.954;.993) | .972(.930;.989) | .946(.864;.979) |
| p-value: *= p ≤0.05, **= p ≤.01 , n.s.= p > .05 (non-significant), Mean: mean of measurements in mm, SD: standard deviation in mm, Mean_diff_: mean difference of measurements in mm, SD_diff_ : difference of standard deviation in mm, SEM: standard error of the mean, MDC: minimal detectable change, ICC: Intraclass correlation coefficient | | | | |

| **Table A4 – Inter- and intraobserver reproducibility for clinical FPI measurements** | | |
| --- | --- | --- |
|  | **DAY1 vs DAY2** | **DAY 1** |
|  | **Intraobserver** | **Interobserver** |
|  |  |  |
| **p-value** | n.s. | * |
| **Mean** | 4.45 | 4.27 |
| **SD** | 2.67 | 2.71 |
| **Mean_diff_** | .70 | 1.10 |
| **SD_diff_** | 1.81 | 1.92 |
| **SEM** | 1.36 | 1.28 |
| **MDC** | 3.55 | 3.76 |
| **ICC** | .875(.684;.950) | .875(.683;.950) |
| p-value: *=p≤0.05, n.s.= p > .05 (non-significant), Mean: mean of measurements in mm, SD: standard deviation in mm, Mean_diff_: mean difference of measurements in mm, SD_diff_ : difference of standard deviation in mm, SEM: standard error of the mean, MDC: minimal detectable change, ICC: Intraclass correlation coefficient | | |

**APPENDIX B – RADIOLOGICAL INTRAOBSERVER MEASUREMENTS**

**Figure B1 – Bland-Altman plot of intraobserver measurements of WB plantar position**


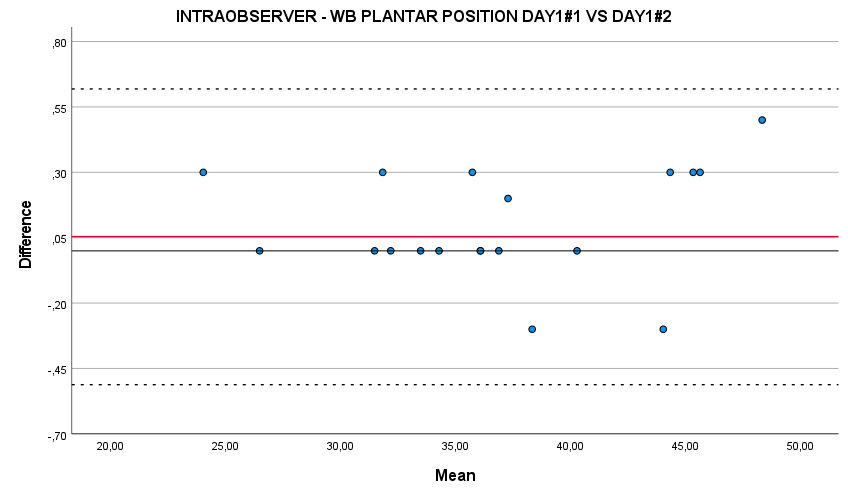
 **Figure B2 – Bland-Altman plot of intraobserver measurements of NWB plantar position**


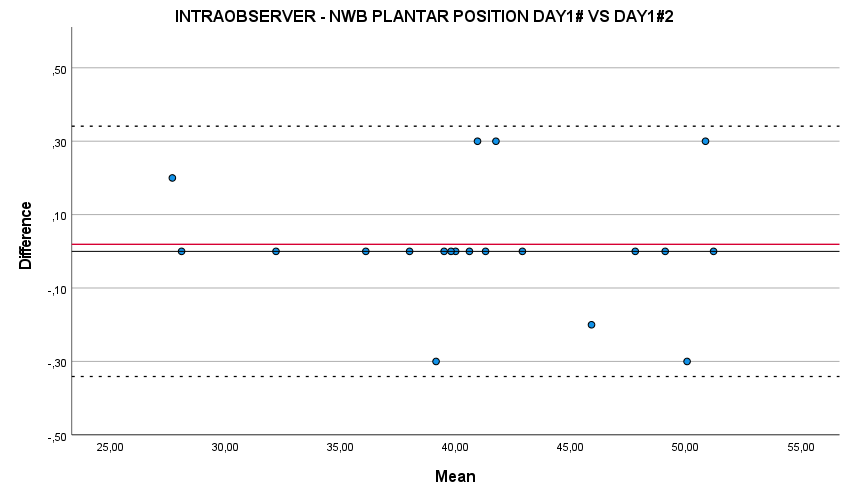


**Figure B3 – Bland-Altman plot of intraobserver measurements of WB medial position**


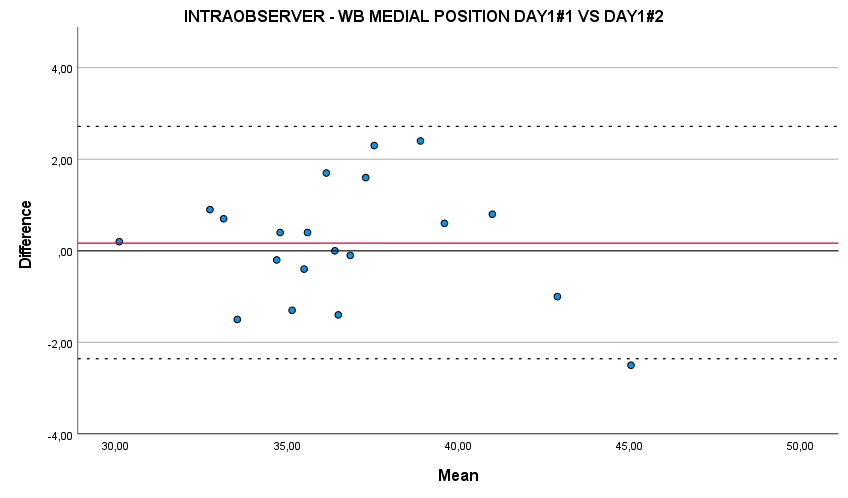


**Figure B4 – Bland-Altman plot of intraobserver measurements of NWB medial position**


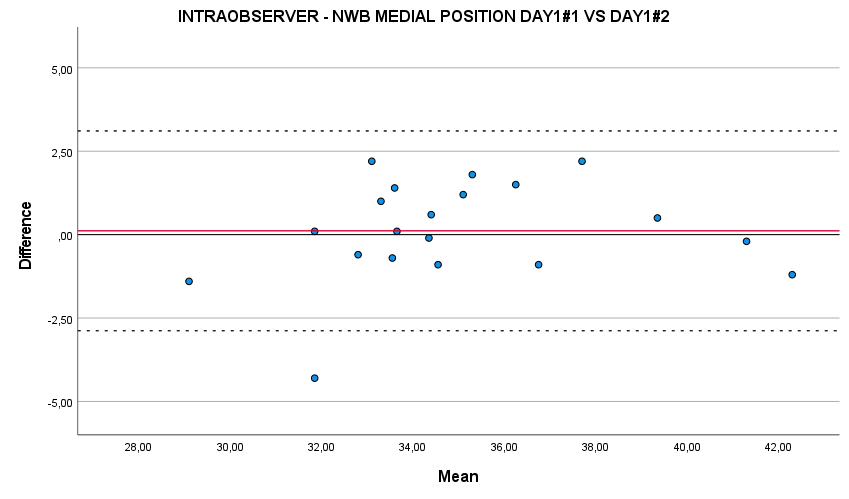


**APPENDIX C – RADIOLOGICAL INTEROBSERVER MEASUREMENTS**

**Figure C1 – Bland-Altman plot of interobserver measurements of WB plantar position day 1**


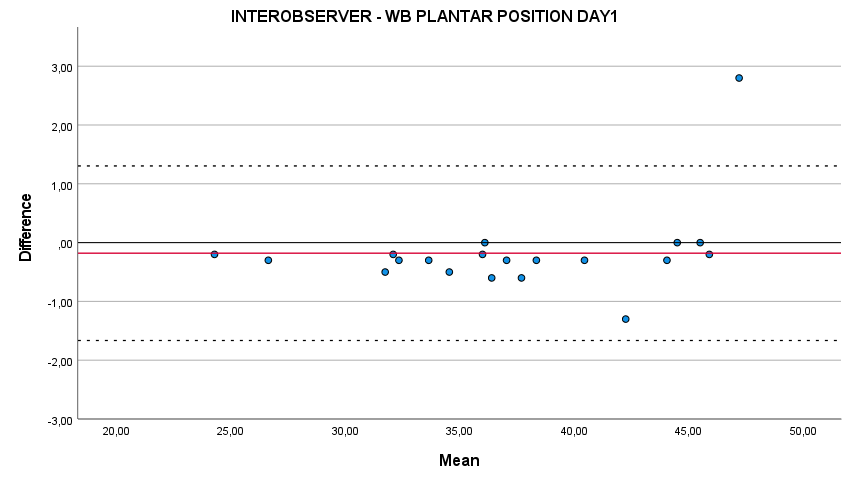


**Figure C2 – Bland-Altman plot of interobserver measurements of WB plantar position day 2**


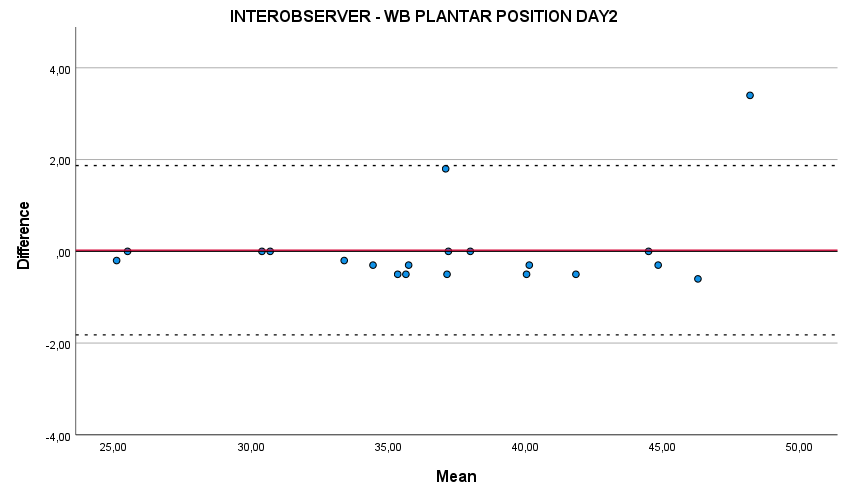


**Figure C3 – Bland-Altman plot of interobserver measurements of NWB plantar position day 1**


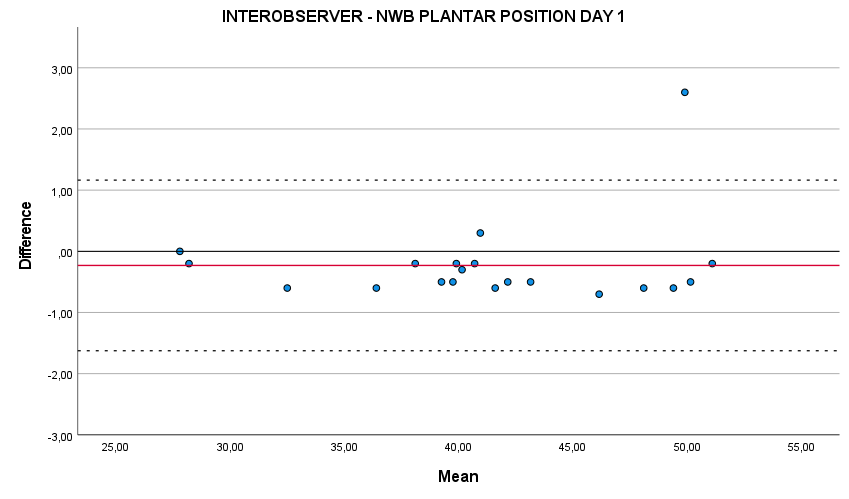


**Figure C4 – Bland-Altman plot of interobserver measurements of NWB plantar position day 2**


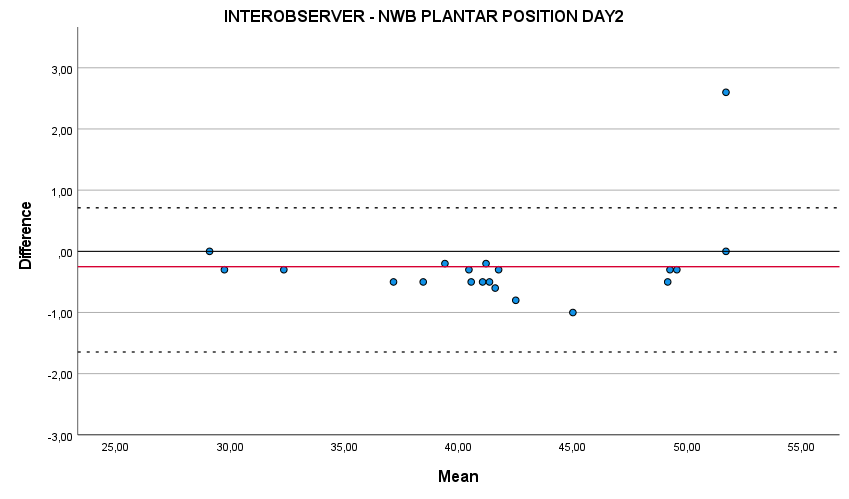


**Figure C5 – Bland-Altman plot of interobserver measurements of WB medial position day 1**


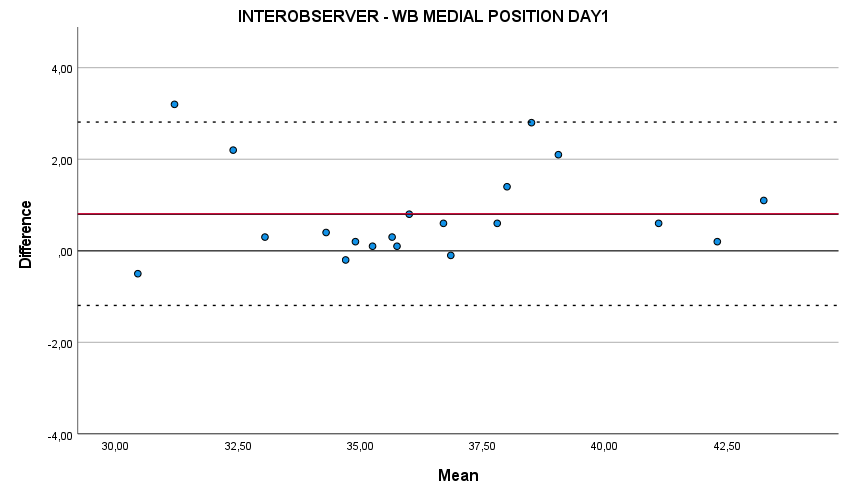


**Figure C6 – Bland-Altman plot of interobserver measurements of WB medial position day 2**


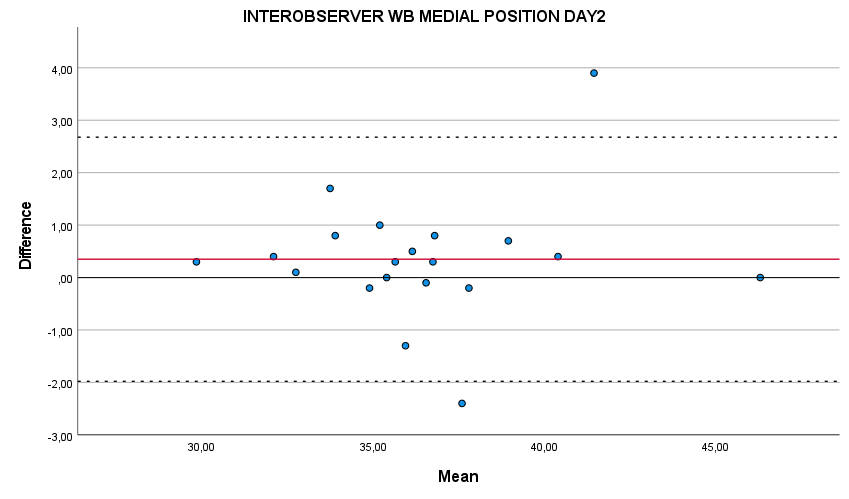


**Figure C7 – Bland-Altman plot of interobserver measurements of NWB medial position day 1**


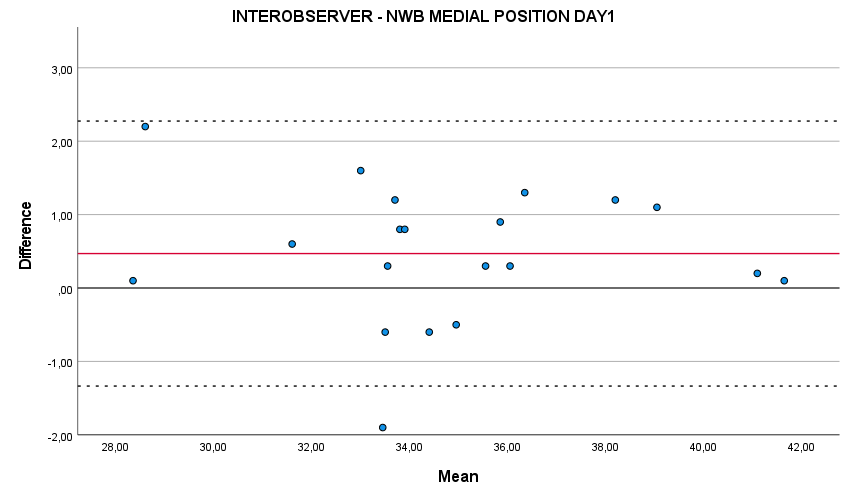


**Figure C8 – Bland-Altman plot of interobserver measurements of NWB medial displacement day 2**


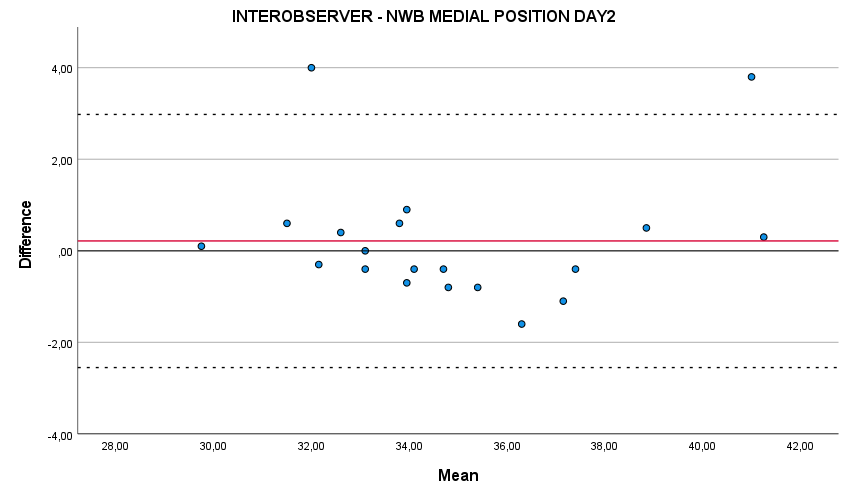


**APPENDIX D – DELTA VALUES BETWEEN WB AND NWB POSITION**

**Figure D1 - Bland-Altman plot of interobserver measurements of plantar displacement delta values between WB and NWB position, between day 1 vs day 2**


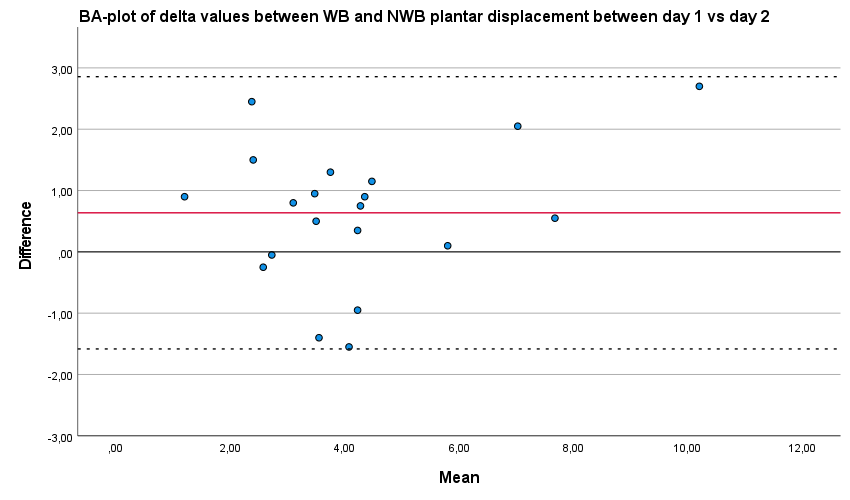


**Figure D2 - Bland-Altman plot of interobserver measurements of medial displacement delta values between WB and NWB position, between day 1 vs day 2**


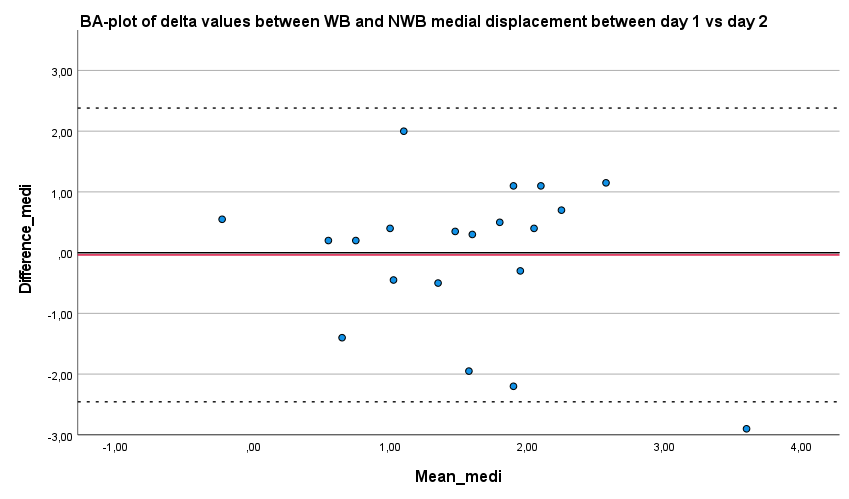


**APPENDIX E – CLINICAL FPI MEASUREMENTS**

**Figure E1 – Bland-Altman plot of intraobserver measurements of total score of clinical FPI measurements day 1 to day 2**


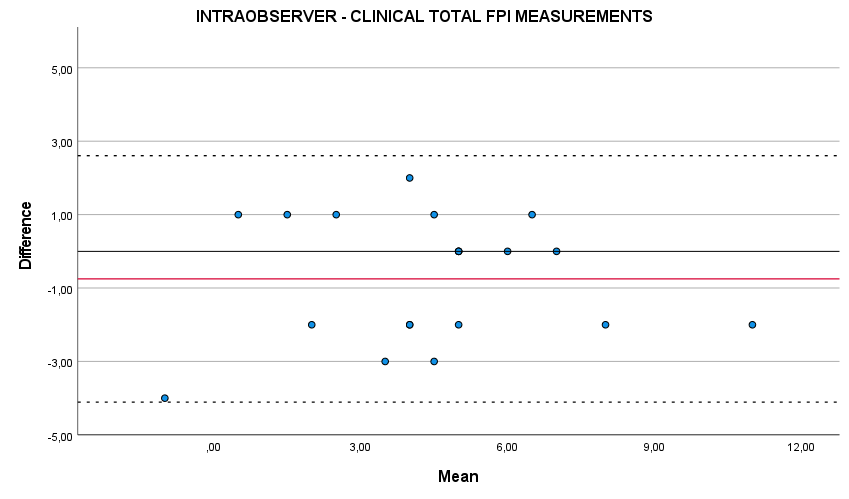


**Figure E2– Bland-Altman plot of interobserver measurements of total score of clinical FPI measurements day 1**


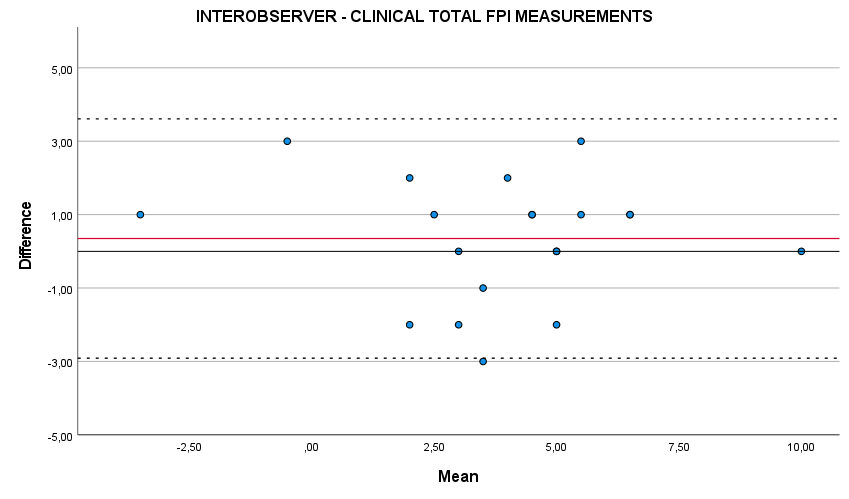

Supplement: Supplementary file 1 — (DOCX 226 kb) [file 256_2023_4352_MOESM1_ESM.docx]
